# A randomized controlled trial of acupuncture to treat functional constipation: design and protocol

**DOI:** 10.1186/1472-6882-14-423

**Published:** 2014-10-29

**Authors:** XiaoHu Xu, CuiHong Zheng, MingMin Zhang, Wei Wang, GuangYing Huang

**Affiliations:** Institute of Integrated Traditional Chinese and Western Medicine, Tongji Hospital, Tongji Medical College, Huazhong University of Science and Technology, 1095 Jiefang Avenue, Wuhan, Hubei 430030 People’s Republic of China; Department of Integrated Traditional Chinese and Western Medicine, Tongji Hospital, Tongji Medical College, Huazhong University of Science and Technology, 1095 Jiefang Avenue, Wuhan, Hubei 430030 People’s Republic of China; Department of Neurology, Tongji Hospital, Tongji Medical College, Huazhong University of Science and Technology, 1095 Jiefang Avenue, Wuhan, Hubei 430030 People’s Republic of China

## Abstract

**Background:**

Functional constipation (FC) is a common functional gastrointestinal disorders (FGIDs) which has a major impact on the quality of life. Acupuncture is widely used as an alternative and complementary medicine (CAM) for FC, but the available evidence of its effectiveness is scarce. Therefore, we will perform a randomized controlled trial to determine whether acupuncture improves symptom and quality of life in FC patients more effectively than sham acupuncture or gastrointestinal prokinetic agent. This article will report the protocol of the trial.

**Methods:**

The current trial is a multicenter, randomized, three-arm controlled study undergoing in China. About 243 people who aged from 18 to 65 years with FC will be recruited in this study. These participants will be randomly allocated into three treatment groups, including electro-acupuncture (EA), Mosapride (M) and Mosapride & Sham Electro-acupuncture (MS) groups in a 1:1:1 ratio. Both the EA and sham EA receives 16 sessions of needling at Quchi (LI11) and Shangjuxu (ST37) during 4 weeks of treatment, and a follow-up period of 4 weeks. These groups will be compared on the primary outcomes of the number of times of defecation at baseline and 2, 4, 8 weeks after randomization. The secondary outcome measures include: stool consistency, intensity of defecating difficulty, MOS item Short Form health survey (SF-36), Self-Rating Anxiety Scale (SAS), Self-rating Depression Scale (SDS), and the validated Patient Assessment of Constipation Quality of Life (PAC-QOL). These outcomes are measured at baseline and 2, 4 weeks after randomization, but SF-36 is measured at baseline and 4 weeks after randomization.

**Discussion:**

This study will supply significant evidence for using acupuncture to treat FC, and will help us to observe whether it is a therapeutic effect rather than a placebo effect.

## Background

Functional constipation (FC) is a common functional gastrointestinal disorders (FGIDs), defined as infrequent bowel movements, hard or lumpy stools, straining during defecation that does not meet the IBS criteria [1]. According to the survey, about 12%-19% population in the US and 14% in Asia suffer from the symptoms of functional constipation [2–4]. As a major public health issue, functional constipation negatively affects health-related quality of life and is associated with substantial costs.

Usual care involves lifestyle modification such as dietary changes and aerobic activities are recommended to help in relieving symptoms but some studies have suggested that the effect is not obviously [5, 6]. In recent years, conventional treatments include gastrointestinal prokinetic agent, enemas, osmotic and stimulant laxatives has displayed a positive effect in functional constipation therapy. However, the efficacy is limited and unwanted side effects are induced with a long therapeutic course [7].

More and more people have focused on alternative and complementary medicine (CAM) to look for a safe and effective therapy for functional constipation in Western countries [8]. Acupuncture as one of the most frequently applied methods in Traditional Chinese Medicine (TCM), which has a history of more than 3000 years in treating functional constipation, has gained increased popularity. Previous studies have shown that acupuncture may be effective in the treatment of functional constipation but have not provided conclusive evidence of its efficacy. An appropriate trial protocol is needed to investigate the effectiveness of acupuncture for functional constipation. Thus we designed a multicenter randomized controlled trial to observe and testify the clinical effects of acupuncture for FC. Meanwhile, we aim to address whether acupuncture treatment is the better therapy compared with medication treatment.

## Methods and Design

### Design

This is a multicenter randomized controlled, three-arm trial enrolling both men and women with functional constipation in China. This study will be conducted at Tongji Hospital affiliated to Tongji Medical College, Huazhong University of Science and Technology; Hubei Provincial Hospital of TCM; Hospital of Huazhong University of Science and Technology. Tongji Hospital is the central structure of the study. The participants will be allocated through complete randomization in a 1:1:1 ratio. The randomization sequence will be generated using R2.0 software. A designated researcher will prepare the assignments in opaque envelopes in sequence. The acupuncturists will only know of the group assignment prior to the treatment.

**Figure 1:**
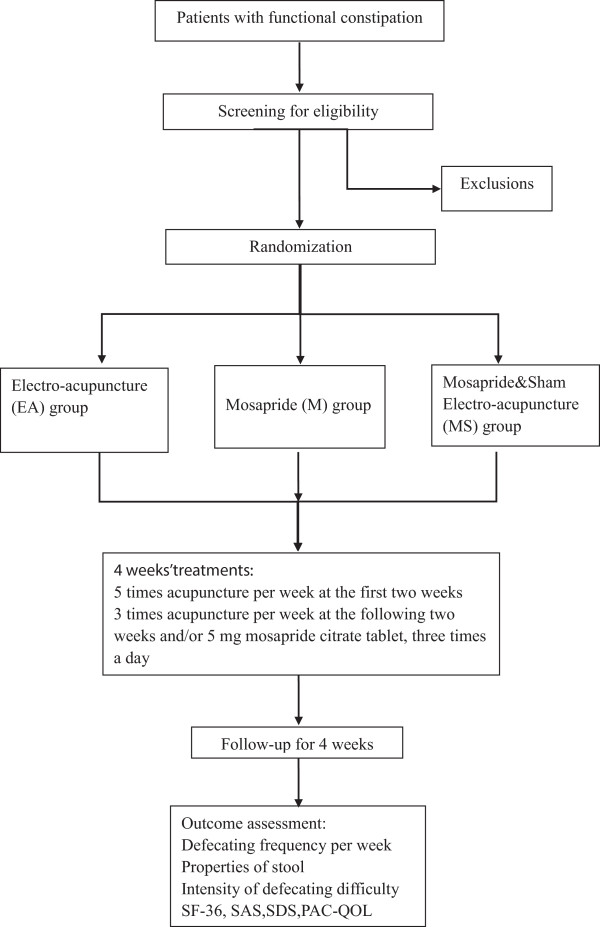
**Trial flow chart.**

This trial is financed by the National Basic Research Program (No. 2011CB505203). The research protocol has been approved by the Clinical Trial Ethics Committee of Tongji Medical College, Huazhong University of Science and Technology (approval no. FWA00007304) and registered on the ClinicalTrials.gov protocol registration system ( http://clinicaltrials.gov/ct2/show/NCT01781897, ClinicalTrials.gov ID: NCT01781897).The duration of this study is 9 weeks for each patient: a screening period of 1 week, a treatment period of 4 weeks, and a follow-up period of 4 weeks. Constipation diaries are required to record at least 1 week before randomization as baseline data and this course will be continue to 8 weeks if they are eligible for this trial. All outcomes are assessed at baseline and 2, 4, and 8 weeks after randomization according to the diaries. A total of 16 sessions of acupuncture treatment will performed over a period of 4 weeks and each session will last 30 min after randomization. Five times a week in the first 2 weeks and three times a week in the last 2 weeks (Figure 1).

## Patients

### Study population

Our study will include patients with chronic functional constipation. To ensure the precision of results of this trial, patients who meet the following eligible criteria will be included in this study.

### Inclusion criteria

Men and women between 18 and 65 years old who met the Rome III Criteria for functional constipation (FC), including two or more of the following symptoms for at least 12 weeks during the 6 months before enrolling the study: (1) straining during at least 25% of defecations; (2) lumpy or hard stools during at least 25% of defecations; (3) sensation of incomplete evacuation during at least 25% of defecations. (4) Sensation of anorectal obstruction or blockage for at least 25% of defecation; (5) Manual maneuvers to facilitate at least 25% of defecation (e.g. support of fingers or pelvic floor); (6) fewer than three spontaneous bowel movements (SBMs) per week. And (1) loose stools are rarely present without the use of laxatives, (2) insufficient criteria for irritable bowel syndrome are necessary. Meanwhile we must confirm all the participants did not use any gastrointestinal drugs at least 1 week before randomization and never joined any other trial in progress. They signed a written consent form and volunteered to finish the whole study.

### Exclusion criteria

Patients with any of the following conditions will be excluded: constipation caused by other endocrine, metabolic, nervous or postoperative diseases or drugs; irritable bowel syndrome (IBS) and inflammatory bowel disease or other structural bowel diseases; psychiatric diseases and cognitive dysfunction or aphasia; tumours and bleeding disorders; serious illnesses of the heart, liver, or kidney, or other severe diseases; pregnant or breastfeeding women; other disorders that may interfere with completion of the study.

### Recruitment procedures

Participants will be recruited in outpatient clinics of hospitals or community hospitals. Participants will also be recruited through local newspaper and the hospital website ( http://www.tjh.com.cn/). Doctors from gastrointestinal surgery and gastroenterology department of the local hospitals would be invited to screen participants.

## Interventions

### Electro-acupuncture (EA) group

The acupuncture treatment bases on traditional Chinese medicine theory and should be operated by licensed acupuncturists. The acupoints are Quchi (LI11) and Shangjuxu(ST37), which are He-Sea and Lower He-Sea acupoints of Large Intestine meridians. Both points were considered as the positive points to treat internal organ’s disease in traditional Chinese medicine (TCM) and were very commonly used in FC patients. Sterile, disposable acupuncture (0.25 mm in diameter and 40 mm in length) are used in this trial, which are purchased from DongBang AcuPrime. Ltd. Co (Marsh Barton, Exeter, UK). In this group, the fixed points will be inserted bilaterally and vertically by filiform needles after sterilizing. A dull needling sensation (which called *de qi* include sour, numb, heavy and aching) will be obtained through lifting, thrusting, twirling and rotating the needles. And then, an electro-acupuncture instrument (HANS-200E, Nanjing Jisheng, made in Jiangsu, China) will connect with each acupuncture needle. The stimulation frequency set 2/50Hz and electric current varies from 0.1 mA to 1.0 mA until the patients feel comfortable. Each session will last for 30 minutes per day. A total of 16 sessions of acupuncture treatment will perform over a period of 4 weeks. Five times a week in the first 2 weeks and three times a week in the last 2 weeks.

### Mosapride (M) group

Participants assigned to this group will receive 5 mg Mosapride Citrate tablet (Dainippon Sumitomo pharmaceutical Co.Ltd, Japan) three times a day for 4 weeks continuously, in the form of an oral capsule administered at least 30 minutes before eating.

### Mosapride & Sham Electro-acupuncture (MS) group

The participants appointed to mosapride & sham acupuncture group will be treated with sham acupuncture while orally given Mosapride Citrate tablets (Dainippon Sumitomo pharmaceutical Co.Ltd, Japan). The dose of mosapride is 5 mg three times a day for 4 weeks continuously as the same as mosapride (M) group. In the sham acupuncture intervention, Park sham devices (PSD) [9] are used, which are purchased from DongBang AcuPrime. Ltd. Co (Marsh Barton, Exeter, UK). It is a non-penetrating needle device with a blunt and retractable needle, a guide tube and a self adhesive pad. The fixed points are bilateral Quchi (LI11) and Shangjuxu (ST37), which are also the same as the treatment group. A precut guide tube which is fixed by a self adhesive pad was slightly depressed down onto the selected point. And then, the blunt sham needle is carefully placed into the guide tube. When pressed, it telescopes into the handle and induces a pricking sensation rather than penetrates the skin. During this process, no twirling lifting and thrusting manipulation is conducted. The sham electro-acupuncture instrument is applied to the needles but the metal inside the wire has been cut off. We set a stimulation frequency of 2/50 Hz and electric current of 0.3 mA but actually, there is no current output and the participants will never know.

### Outcome measures

The primary outcomes of the study is the times of defecation per week at baseline and at the 2nd, 4th and 8th week after randomization. Secondary efficacy end points include: 1) stool consistency [10]; 2) intensity of defecating difficulty; 3) MOS item Short Form health survey (SF-36) [11]; 4) Self-Rating Anxiety Scale (SAS) [12]; 5) Self-rating Depression Scale (SDS) [13]; 6) the validated Patient Assessment of Constipation Quality of Life (PAC-QOL) [14] (Table 1).

**Table 1:** **Trial processes chart**

Week	-1	0	2	4	8
	Baseline	Treatment			Follow-up
**Patients**					
Medical history	√				
Physical examination	√	√	√	√	
Laboratory examination	√			√	
Informed consent		√			
Randomization		√			
Expectation of acupuncture		√			
**Intervention**					
Electro-acupuncture (EA) group	16 sessions of electro-acupuncture				
Mosapride (M) group	5 mg mosapride citrate tablet, three times a day				
Mosapride & Sham	16 sessions of sham electro-acupuncture and 5 mg mosapride citrate tablet, three times a day				
Electro-acupuncture (MS) group					
**Outcomes**					
Number of defecating events per week		√	√	√	√
Shape and properties of stool		√	√	√	√
Intensity of defecating difficulty		√	√	√	√
SF-36		√		√	
SAS		√	√	√	
SDS		√	√	√	
PAC-QOL		√	√	√	√
**Trial evaluation**					
Safety of electro-acupuncture		√	√	√	
Adverse event		√	√	√	
Reasons of drop-outs or withdrawals				√	
Patient’s compliance				√	

SF-36, MOS item Short Form health survey; SAS, Self-Rating Anxiety Scale; SDS, Self-rating Depression Scale; PAC-QOL, patient assessment of constipation quality of life questionnaire.

The first three outcomes will be measured at the other time points (baseline and 2, 4, 8 weeks after randomization) according to the daily records written by participants. Stool consistency was assessed by the Bristol Stool Form Scale (BSFS) [10]. Intensity of defecating difficulty was assessed by a 4-point scale with the following responses: 0 indicates not at all, 1 a little bit, 2 a moderate amount, 3 a great deal and an extreme amount. PAC-QOL include 28 items related to the effects of constipation on daily lives. These outcomes will be measured at baseline and 2, 4, 8 weeks after randomization which provided by participants. Patients are asked to complete the SAS and SDS questionnaire at baseline and 2 and 4 weeks after randomization. SF-36 questionnaire is completed before randomization and 4 weeks after randomization.

Before randomization all patients will receive routine tests of urine, stool, blood biochemical (ALT, AST, BUN, and Scr), electrocardiogram (ECG), and colonoscopy to exclude these patients who have other severe diseases. Urine HCG or blood β-HCG will also be tested to exclude possible pregnant women. These routine tests should also be done after accomplishment of intervention to help us assess the adverse events.

Adverse events including bleeding, hematoma, fainting, serious pain, and local infection should be recorded and resolved immediately during all duration of this study. Serious adverse events will be documented and immediately reported to the principal investigator.

### Sample size calculation

Sample size was determined based on a study [15] where the average defecation rate of patients with functional constipation was 2.6 times per week with a standard deviation of 2.2 after drug treatment, and a 1.4-fold difference in the clinical effects between the drug and the placebo is considered to be of clinical relevance. In this study, we anticipated that an improvement of the mean defecating frequency is 4.0 after acupuncture treatment, with a standard deviation of 3. To detect this difference, we need a sample size of 71 in each group (α = 0.05, β = 0.10) [16]. To allow 15% for loss to follow up, our proposed trial requires 243 patients (81 for each group).

### Statistical analysis

Statistical analysis of all data will be done by the Department of Epidemiology and Biostatistics, School of Public Health, Tongji Medical College, Huazhong University of Science and Technology, which is blinded to this trial. SPSS 17.0 software packages will be used to analyze the data. All data are provided as mean ± standard deviation (SD) in continuous data and as frequency/percentage in categorical data. All of the analyses will be based on both an intention-to-treat analysis (ITT) and a treated-per-protocol analysis (TPP). The intention-to-treat (ITT) population was defined as the patients who are randomized and received at least one treatment session. The missing data will be dealt with the last observation value carried forward (LOCF) method. The per-protocol (PP) population was defined as the patients who have no major protocol violations in the ITT set. Both results of analysis will be compared to check whether the results are consistent.

Comparisons among baseline characteristics in different groups will be analyzed at first by analysis of variance, Chi-square test, and others. Then analysis will be applied to compare the effects of intervention between groups. Chi-square test will be used for comparison of categorical variables and analysis of variance of repeated measures will be performed for continuous parameters between the groups. The level of significance was set a P value less than 0.05.

### Quality control

In order to guarantee the quality of trial, all acupuncturists are asked to attend special training. The training includes how to fill in the case report form, how to manipulate both true and sham needles, how to teach the patients to take Mosapride Citrate tablets, and other details of this trial. A special training test will be given to the acupuncturists to ensure their understanding of protocol. After passing the test they are qualified to perform this trial. Only acupuncturists will know the grouping scheme and all the researchers are forbidden to alter the assignment. Inspectors from the central structure of the study will check each research center monthly and report to the investigator. Reasons and details of drop-outs or withdrawals will be documented.

## Discussions

According to TCM theory, the body was regarded as a whole composed of internal and external organs. These organs connected with each other by special ways called meridians, in which "qi" and "blood" is circulating. Disease is a state that "qi" and "blood" is deviated from balance. Functional constipation could be attributed to "defecation difficulty" in traditional Chinese Medicine (TCM), which were considered to be caused by improper diet, emotional disturbance, and invasion of exogenous pathogen. According to the different pathogenic factors, "defecation difficulty" could be classified into the categories of "Qi-deficiency constipation", "Blood-deficiency constipation", "Yin-deficiency constipation", and "Qi-deficiency constipation" etc. These disturbances attributed to dysfunction of large intestinal transit function and insufficient large intestinal liquid.

Chinese medicines, acupuncture and moxibustion as effective complementary and alternative medicine (CAM) have been widely used to treat FC in China and clinically adopted in the western world [17]. Acupuncture has been believed to treat FC at least 3000 years. Some studies have indicated that acupuncture can play a therapeutic role in Functional gastrointestinal (GI) symptoms through alter acid secretion, GI motility, and visceral pain [18–21]. However, the literature which has been made to investigate the efficacy of acupuncture on function constipation is scarce. Some pilot studies reported that acupuncture could improve symptoms of constipation but did not set a control group [22, 23]. In another study, acupuncture has been found to increase the frequency of bowel movement in 17 children with chronic constipation during a 10-week treatment period but not include adult patients [24]. Based on the current evidences from clinical trials, acupuncture seems show potential for treating constipation. However, this deduction is insufficient and may lead to bias because the poor quality without a standardized acupuncture protocol. Therefore we designed a multicenter randomized controlled protocol and expected to provide convincing evidence to clarify the efficacy of acupuncture for treating FC.

Acupoints selected in this trial were Quchi (LI11) and Shangjuxu (ST37), which were He-Sea and Lower He-Sea points of Large Intestine meridians. According to traditional acupuncture theories, both of them have a positive therapeutic effect in treating internal organ disease. Differing from the other studies adopted non-acupoints in placebo control, we will use a validated placebo needling [9] and special electrode lines with no current output at the same acupoints to mimic as closely as possible the real situation on patients. In the other hand, to avoid communication with each other, patients will be assigned to acupuncture treatments alone at different times. In addition, we will use mosapride as a positive control group because it has been confirmed as an effective drug to treat FC in China and western countries. The primary outcome assessment will be collected from the diaries which will be convenient and easy for patients to record. Certainly, the safety of interventions is needed to consider during the whole process.

As far as we know, this is the first acupuncture trial adopting sham EA combined with mosapride (MS) as a control to observe the efficacy of acupuncture for treating FC. The reasons we selected mosapride & sham acupuncture as a control instead of simple placebo acupuncture based on: Firstly, it will help to improve therapy compliance and reduce the rate of drop-out so as to ensure that the study is completed smoothly, because a big part of patients expect to receive acupuncture treatment when they participate in this trial. Second, whether simple placebo acupuncture is effective for patients with functional constipation is controversial, so it accords with the ethical principles of beneficence and justice to avoid ethical confusion.

However, some limitations we must also recognize that patients are not blinded in this trial. The participants will know whether they receive acupuncture or medication although they will not know true or sham acupuncture they will receive. And then, the physiological effects may be amplified when patients have a higher positive beliefs and expectations of benefit of acupuncture compared with medicine, which will impact the acupuncture effect. These factors will lead to a bias of the outcome assessment. In conclusion, the study will supply significant evidence for using acupuncture to treat FC, and help us to observe whether it is a therapeutic effect rather than a placebo effect.

## Trial status

This trial is currently recruiting participants. The first participant was included on 20 March 2012 and the investigators are still collecting.

## References

[1] Pare P, Ferrazzi S, Thompson WG, Irvine EJ, Rance L (2001). An epidemiologicalsurvey of constipation in Canada:definitions, rates, demographics, and predictors of health care seeking. Am J Gastroenterol.

[2] Higgins PD, Johanson JF (2004). Epidemiology of constipation in North America: a systematic review. Am J Gastroenterol.

[3] Lembo A, Camilleri M (2003). Chronic constipation. N Engl J Med.

[4] Cheng C, Chan AO, Hui WM, Lam SK (2003). Coping strategies, illness perception, anxiety and depression of patients with idiopathic constipation: a population-based study. Aliment Pharmacol Ther.

[5] Meshkinpour H, Selod S, Movahedi H, Nami N, James N, Wilson A (1998). Effects of regular exercise in management of chronic idiopathic constipation. Dig Dis Sci.

[6] Young RJ, Beerman LE, Vanderhoof JA (1998). Increasing oral fluids in chronic constipation in children. Gastroenterol Nurs.

[7] Ford AC, Suares NC (2011). Effect of laxatives and pharmacological therapies in chronic idiopathic constipation: systematic review and meta-analysis. Gut.

[8] van Tilburg MA, Palsson OS, Levy RL, Feld AD, Turner MJ, Drossman DA, Whitehead WE (2008). Complementary and alternative medicine use and cost in functional bowel disorders: a six month prospectivestudy in a large HMO. BMC Complement Altern Med.

[9] Park JB, White A, Lee HJ, Ernst E (1999). Development of a new sham needle. Acupunct Med.

[10] Lewis SJ, Heaton KW (1997). Stool form scale as a useful guide to intestinal transit time. Scand J Gastroenterol.

[11] Li XM, Duan LP, Wan CH, Zhou ZF, Pan JH, Yang Z, Zhang XQ (2006). Evaluation of SF-36 instrument with the quality of life in chronic diseases. China Med.

[12] Liu XC, Tang MQ, Dai XG, Chen K, Dai ZS (1995). Factor analysis of self-rating anxiety scale. Chin J Nervous Mental Dis.

[13] Wang CF, Cai ZH, Xu Q (1986). Evaluation analysis of self-rating depression disorder scale in 1,340 people. Chin J Nervous Mental Dis.

[14] Zhao ZZ, Lin Z, Lin L, Zhang HJ, Wang MF, Wang Y (2010). Quality of life and related factors in patients with chronic constipation. Journal of Nursing Science.

[15] Camilleri M, Kerstens R, Rykx A, Vandeplassche L (2008). A placebo-controlled trial of prucalopride for severe chronic constipation. N Engl J Med.

[16] Xu J, Wang L, Xia J (2009). Sample size estimation and statistical inference in a three-arm non-inferiority clinical trial including a placebo and an active control. Chinese J Health Stat.

[17] Anders EF, Findeisen A, Nowak A, Rüdiger M, Usichenko TI (2012). Acupuncture for treatment of hospital-induced constipation in children: a retrospective case series study. Acupunct Med.

[18] Tougas G, Yuan LY, Radamaker JW, Chiverton SG, Hunt RH (1992). Effect of acupuncture on gastric acid secretion in healthy male volunteers. Dig Dis Sci.

[19] Xu GY, Winston JH, Chen JD (2009). Electroacupuncture attenuates visceral hyperalgesia and inhibits the enhanced excitability of colon specific sensory neurons in a rat model of irritable bowel syndrome. Neurogastroenterol Motil.

[20] Tatewaki M, Harris M, Uemura K, Ueno T, Hoshino E, Shiotani A, Pappas TN, Takahashi T (2003). Dual effects of acupuncture on gastric motility in conscious rats. Am J Physiol Regul Integr Comp Physiol.

[21] Takahashi T (2013). Effect and mechanism of acupuncture on gastrointestinal diseases. Int Rev Neurobiol.

[22] Long ZR, Yu CH, Yang Y, Wang HN, Chi XX (2006). Clinical observation on acupuncture combined with microorganism pharmaceutical preparations for treatment of irritable bowel syndrome of constipation type. Zhongguo Zhen Jiu.

[23] Chan J, Carr I, Mayberry JF (1997). The role of acupuncture in the treatment of irritable bowel syndrome: a pilot study. Hepatogastroenterology.

[24] Broide E, Pintov S, Portnoy S, Barg J, Klinowski E, Scapa E (2001). Effectiveness of acupuncture for treatment of childhood constipation. Dig Dis Sci.

[CR25] The pre-publication history for this paper can be accessed here: http://www.biomedcentral.com/1472-6882/14/423/prepub

